# Factors associated with the success of rabies vaccination of dogs in Sweden

**DOI:** 10.1186/1751-0147-53-22

**Published:** 2011-03-25

**Authors:** Louise T Berndtsson, Ann-Kristin J Nyman, Esteban Rivera, Berndt Klingeborn

**Affiliations:** 1 National Veterinary Institute, Uppsala, Sweden

## Abstract

**Background:**

United Kingdom, Ireland, Malta and Sweden maintain their national provisions for a transitional period regarding rules concerning rabies vaccination and individual serological test for rabies neutralizing antibodies. The purpose of vaccinating dogs against rabies is to establish pre-exposure immunity and protect individual animals from contracting rabies.

The aim of the study was to investigate factors associated with reaching the internationally accepted threshold antibody titre of 0.5 IU/mL after rabies vaccination of dogs.

**Methods:**

The study was a prospective single cohort study including 6,789 samples from Swedish dogs vaccinated with commercially available vaccines in Sweden, and the dog's antibody responses were determined by the OIE approved FAVN test. Information on potential risk factors; breed, age, gender, date of vaccination, vaccine label and the number of vaccinations, was collected for each dog. Associations between the dependent variable, serological response ≥ 0.5 IU/mL or < 0.5 IU/mL and each of the potential risk factors were investigated using logistic regression analysis.

**Results:**

Of 6,789 vaccinated dogs, 6,241 (91.9%) had an approved test result of ≥ 0.5 IU/mL. The results of the multivariable logistic regression analysis showed that vaccinating with vaccine B reduced the risk of having antibody titres of < 0.5 IU/mL by 0.2 times compared with vaccination using vaccine A. Breed size was found significant as an interaction with number of vaccinations and age at vaccination as an interaction with day of antibody testing after last vaccination. In summary, larger breeds were at higher risk of having antibody titres of < 0.5 IU/mL but if vaccinated twice this risk was reduced. Moreover, there were a increased risk for dogs < 6 months of age and > 5 years of age to have antibody titres of < 0.5 IU/mL, but this was affected by number of days from vaccination till testing.

**Conclusions:**

The probability of success of rabies vaccinations of dogs depends on type of vaccine used, number of rabies vaccinations, the breed size of the dog, age at vaccination, and number of days after vaccination when the antibody titres are tested. The need for a booster vaccination regimen is recommended for larger breeds of dog.

## Background

Sweden is free from rabies since more than 100 years. When Sweden joined the European Union 1994 the obligatory quarantine system was abandoned and new rules for non-commercial movement of pet animals (dogs, cats and ferrets) were put in place. The rules are laid down in Directive 998/2003 of the European Community [1]. According to these rules all animals should be identified by tattoo and/or microchip and vaccinated against rabies, and a 21-day waiting period in case of primary vaccination. In addition, article 6 of the above Regulation provides that four countries, United Kingdom, Ireland, Malta and Sweden, maintain their national provisions for a transitional period. For Sweden these derogations consist of the requirement of an individual serological test for rabies neutralizing antibodies before entry into Sweden earliest 120 days after the latest vaccination.

The purpose of vaccinating cats and dogs against rabies is to establish pre-exposure immunity and protect individual animals from contracting rabies, hereby preventing further spread to humans or other domestic animals.

It has been shown by others that type of vaccine used, number of vaccinations, interval between vaccination and blood sampling, age at vaccination, size and breed can influence the antibody response [2-5], but this has not been investigated in Sweden.

The aim of the study was to investigate factors associated with reaching the internationally accepted threshold antibody titre of 0.5 IU/mL after rabies vaccination of dogs.

## Methods

### Study design

The study was a prospective single cohort study including 6,789 blood samples from dogs received by the National Veterinary Institute (SVA) during 2005 for the analysis of the serological response to rabies vaccination. The samples were either whole blood samples or sera from dogs vaccinated with one of the two commercially available vaccines in Sweden. To qualify into the study the dogs had to be vaccinated in Sweden and been vaccinated against rabies with one of the two in Sweden commercially available vaccines, and samples for serological analyses had to be sent in to SVA.

The samples were collected as routine samples, hence, not especially for this study. Owners were informed at sampling that the sample could be used for research and they were given the opportunity to not let the sample be used for research.

### Vaccines

Two, in Sweden commercially available monovalent inactivated rabies vaccines, were evaluated. A: Nobivac^® ^Rabies Vet. (Intervet AB) and B: Rabisin^® ^Vet. (Merial Norden A/S). From here on these vaccines are referred to as vaccines A and B, respectively. Vaccine A contains ≥ 2 IU of the rabies virus strain RIV (Pasteur Institute) per vaccine dose plus aluminum phosphate as adjuvant and vaccine B contains ≥ 1 IU of rabies virus Wistar G 57 (Pasteur Institute) and aluminum hydroxide as adjuvant. The main task of adjuvants is to induce an inflammatory response which is needed for an adaptive immune response including T and B cells. Also adjuvant can be important for constituting depot effect enabling a sustained presence of the vaccine antigen in the body, Adjuvants can also be important for the need of less vaccine antigen in an adjuvanted vaccine. The dogs were immunized following the vaccine producers' instructions.

### Sample collection

Blood samples were collected throughout the country at different veterinary clinics. The samples were collected 120 days post vaccination up to 360 days post vaccination. The samples from the dogs included in this study were accompanied by referrals containing breed, age, gender, date of vaccination, vaccine label and the number of vaccinations. Of the 6,789 dogs included in the study 3,571 (52.6%) received vaccine A and 3,218 (47.4%) dogs received vaccine B.

### Serological analysis

All the samples were analyzed at the SVA. The antibody responses were determined by the OIE approved FAVN test [6]. Dogs with titres of ≥ 0.5 IU/mL were considered to have passed the test and dogs with titres of < 0.5 IU/mL were considered to have failed the test. An antibody titre of ≥ 0.5 IU/mL is the international accepted threshold after rabies vaccination of dogs.

### Statistical analysis

Associations between the dependent variable, serological response ≥ 0.5 IU/mL or < 0.5 IU/mL and each of the potential risk factors; type of vaccine, day of sampling after last vaccination, number of vaccinations, age at vaccination, breed size, pure-bred or not, and gender were first investigated using univariable logistic regression analysis. Before the regression analysis the linear association between age at vaccination and the dependent variable was investigated on a logistic scale, and was found not linear, hence, age at vaccination was categorized into 5 approximately evenly sized categories. Pure-bred dogs were categorized according to size, using information on the website http://www.svenskhund.se/hund_raspresentation.asp?val=lista&sort=storlek (in Swedish) where breed sizes, based on the definitions in the breeding standards, are categorized into four categories; very small - small (< 30 cm in height), small - medium (30-45 cm in height), medium - large (45-60 cm in height), and large - very large (> 60 cm in height). There was no information about the height of dogs of mixed breed so they were categorized as dogs of unknown size (only including mixed breeds) in the breed size variable. All variables, provided that there was no collinearity (r < 0.70) between variables, were then considered for the multivariable analysis. Collinearity between variables was assessed pair-wise by calculation of Spearman rank correlations. A multivariable model was constructed using manual stepwise backwards regression analysis, where variables not significant in the model were re-entered whenever a new variable became significant, or a variable was removed. Potential confounders were considered, and a variable was considered as a confounder if the point estimates of the coefficients in a model change > 20% with the potential confounder present. In the final model a variable with a *P*-value ≤ 0.05 was considered statistically significant and retained in the model. Biologically plausible interactions between the main effects were tested in the final model.

Model validation was preformed according to Hosmer and Lemeshow [7]. The fit of the model was evaluated with the Hosmer-Lemeshow goodness-of-fit test with the data partitioned into 10 deciles, and by visual examination of diagnostic plots. Plot of Pearson residuals (r), leverage (*h*), delta beta (Δβ), delta deviance (ΔD), and delta chi^2 ^(Δχ^2^) versus the predicted values were constructed and evaluated. Observations with divergent values, i.e. -3 ≤ r ≥ 3, *h *> 0.3, Δβ > 1, ΔD > 4.0, or Δχ^2 ^> 4.0 were considered outliers. The impact of outliers was assessed by running the model without the observations considered as outliers, and comparing the coefficients between this model and the model using all observations. Data editing and all the statistical analyses were performed in Stata Software (StataCorp., 2003; Stata Statistical Software: Release 10.0; College Station, TX, USA: StataCorp LP.).

## Results

### Descriptive data and univariable analysis

Descriptive data of the dependent variable and the risk factors investigated, and their *P*-value in the univariable analysis are presented in Table 1. Of 6,789 vaccinated dogs, 6,241 (91.9%) had an approved test result of ≥ 0.5 IU/mL. There were 3,571 dogs vaccinated using vaccine A, and 3,218 dogs vaccinated using vaccine B. In the univariable analysis it was shown that dogs vaccinated with vaccine B more often reached approved antibody titres than dogs vaccinated with vaccine A (*P *< 0.001). Moreover, significantly more dogs reached approved test results if antibody titres were checked at day 120-150 after vaccination compared to if they were checked at day 151-180 (*P *< 0.004), and two immunizations significantly increased the number of dogs reaching approved test results (*P *< 0.001). Dogs < 6 month and ≥ 5 years more often had less success in reaching approved test result compared with dogs between 6 months < 5 years of age (*P *< 0.05). Breed size and breed was also significantly associated with approved test result in the univariable analysis; smaller dogs and dogs of mixed breed were more likely to reach approved test results compared with larger dogs and pure-bred dogs (*P *< 0.05).

**Table 1:** Distribution of potential risk factors associated with the success (antibody titres ≥ 0.5 IU/ml) of rabies vaccination in dogs (n = 6,789).

Variable	Level	Number of animals	Proportion of dogs with antibody titres ≥ 0.5 IU/ml, %	P-value in the univariable logistic regression analysis
**Type of vaccine**	1:Vaccine A	3571	87.4	
	2:Vaccine B	3218	96.9	<0.001
**Day of antibody **testing after last vaccination	1: 120 - 150 days	5156	92.6	
	2: 151 - 180 days	1613	90.3	0.003
**Number of **vaccinations	1: Once	1766	85.7	
	2: Twice	5023	94.1	< 0.001
**Age at vaccination**	1: < 6 month	1635	89.5	
	2: 6-11.9 months	1050	92.6	
	3: 1-2.49 years	1692	93.8	
	4: 2.5 - 4.99 years	1053	92.6	
	5: ≥ 5 years	698	90.4	< 0.001
**Breed size**	1: Very small/small pure-breed (< 30 cm in height)	1482	94.1	
	2: Small/medium sized pure- breed (30-45 cm in height)	1203	92.2	
	3: Medium/large pure-breed (46-60 cm in height)	1965	91.4	
	4: Large/very large pure-breed (> 60 cm in height)	1345	88.4	
	5: Unknown size mixed breeds	747	94.5	< 0.001
**Gender**	1: Bitch	3637	91.4	
	2: Dog	3152	92.5	0.12

### Multivariable analysis

**Figure 1:**
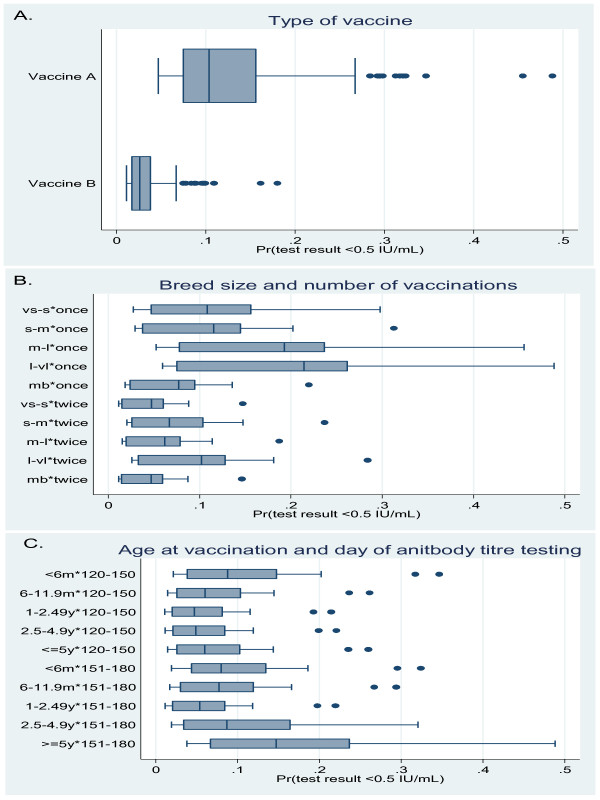
**Distribution of the probability of having antibody levels of < 0.5 IU/ml for all main effects and interactions**. **A**. Type of vaccine used. **B**. Interaction between breed size^1 ^and number of vaccinations. **C**. Interaction between age^2 ^at vaccination and time of antibody titre testing after the latest vaccination in the final multivariable logistic regressions analysis of variables associated with the success of rabies vaccinations in 6,071 Swedish dogs. ^1^vs-s = very small - small breed; s-m = small - medium breed; m-l = medium - large breed; l-vl = large to very large breed. ^2^Age in month (m) or year (y).

Of all the 6 variables considered in the multivariable analysis only gender was not retained in the final model. In the final model only type of vaccine remained as a main effect while breed size was found significant as an interaction with number of vaccinations and age at vaccination as an interaction with day of antibody testing after last vaccination (Table 2 and Figure 1). The results show that vaccinating with vaccine B will reduce the risk of having antibody titres of < 0.5 IU/mL by 0.2 times (i.e. if 10% of the dogs vaccinated with vaccine A fail to reach an antibody titre of ≥ 0.5 IU/mL, only 2% of the dogs would have failed if vaccinated with vaccine B instead).

**Table 2:** Final multivariable logistic regression analysis of variables significantly (P ≤ 0.05) associated with success of rabies vaccinations in 6,071 Swedish dogs (pseudo R^2 ^= 0.11).

Variable	*β*	S.E.(*β*)	OR^a^	95% CI^b ^(OR^a^)	*P*-value
In**tercept**	-1.44	0.19	-	-	-
Vaccine					
**A: Nobivac**	Ref	-	-	-	-
**B: Rabisin**	-1.47	0.12	0.23	0.18, 0.29	< 0.001
***Interactions***					
Breed size * no of vaccin**ations**					
Very small -small breed size * vaccinated once	Ref	-	-	-	-
**Small **- medium bred size * vaccinated once	0.07	0.27	1.07	0.63, 1.84	0.79
**Medium **- large breed size * vaccinated once	0.68	0.21	1.97	1.29, 3.00	0.002
**Large **- very large breed size * vaccinated once	0.81	0.22	2.25	1.45, 3.49	< 0.001
Unknown size (mixed breed) * vaccinated once	-0.41	0.38	0.66	0.32, 1.39	0.28
Very small -small breed size * vaccinated twice	-0.90	0.24	0.41	0.25, 0.65	< 0.001
**Small **- medium bred size * vaccinated twice	-0.31	0.22	0.73	0.47, 1.13	0.16
**Medium **- large breed size * vaccinated twice	-0.61	0.21	0.54	0.36, 0.82	0.004
**Large **- very large breed size * vaccinated twice	-0.07	0.21	0.93	0.62, 1.42	0.75
Unknown size (mixed breed) * vaccinated twice	-0.91	0.29	0.40	0.23, 0.72	0.002
Age at vaccination * number of day after **vaccination a.b. titres were tested**					
< 6 month * day 120-150	Ref	-	-	-	-
6-11.9 month * day 120-150	-0.40	0.17	0.67	0.48, 0.93	0.018
1-2.49 years * day 120-150	-0.67	0.16	0.51	0.38, 0.70	< 0.001
2.5-4.99 years * day 120-150	-0.63	0.18	0.53	0.38, 0.75	< 0.001
≥ 5 years *day 120-150	-0.41	0.19	0.66	0.45, 0.96	0.032
< 6 month * day 151-180	-0.10	0.20	0.90	0.60, 1.35	0.62
6-11.9 month * day 151-180	-0.24	0.25	0.78	0.48, 1.29	0.34
1-2.49 years * day 151-180	-0.63	0.22	0.53	0.34, 0.82	0.004
≥ 2.56 years * day 151-180	-0.12	0.24	0.89	0.56, 1.42	0.62
≥ 5 years *day 151-180	0.58	0.25	1.80	1.10, 2.93	0.019

^a^OR = odds ratio

^b^CI = confidence interval

Medium to large and large to very large pure-bred dogs were at increased risk of having antibody titres of < 0.5 IU/mL compared to very small to small pure-bred dogs or mixed breed dogs of unknown size when vaccinated once. However, if medium to large and large to very large pure-bred dogs were vaccinated twice compared to once, the risk of having antibody titres of < 0.5 IU/mL were reduced. For pure-bred dogs of small to medium size and for dogs of mixed breed of unknown size there was no significant difference in risk of having antibody titers < 0.5 IU/mL. Of dogs vaccinated twice there were an increased risk for small to medium and large to very large pure-bred dogs to have antibody titres of < 0.5 IU/mL vaccinated compared to very small to small pure-bred dogs and mixed breed dogs of unknown size.

Dogs at an age ≥ 6 months at vaccination had a decreased risk of having antibody titres < 0.5 IU/mL than dogs < 6 month of age at vaccination when antibody levels were tested at day 120-151 after last vaccination. There was no difference in risk between dogs ≥ 6 months tested at day 120-151. For dogs ≥ 5 years there were an increased risk of having antibody titres < 0.5 IU/mL if the antibody level was tested at day 151-180 compared with if the antibody level was tested at day 120-150 after last vaccination. For dogs < 5 years there was no difference in risk if the antibody level were tested at day 120-150 or 151-180 days after vaccination. Dogs at the age of 1-2.49 years at vaccination that were tested for antibody levels at day 151-180 had a reduced risk of having antibody titres < 0.5 IU/mL compared to dogs < 6 months, and dogs of ≥ 5 years of age at the time of vaccination, that were tested at day 151-180. There was no significant difference in risk between dogs of other ages tested at day 151-180.

### Model fit

The final model showed good fit; the Hosmer-Lemeshow χ^2 ^(8 d.f.) was 6.42 (*P *= 0.60). When looking at different plots of r, *h*, Δβ, ΔD and Δ χ^2^, several divergent covariates were seen (n = 2-8, dependent on which diagnostic value was addressed), but the coefficients did not change considerably and the model did not improve much with deletion of the divergent observations.

## Discussion

This study comprised 6,789 blood samples from dogs analyzed for the serological response to rabies vaccination. An important outcome of the study was the significant difference in failure rates between the two vaccines used in Sweden. This has also been shown by others [2,5,8]. However, the study includes a large number of dogs, which were all tested more than 120 days post vaccination. The majority of dogs were also vaccinated twice, and to the best of our knowledge there is no comparative published data on the use of two doses of rabies vaccine and samples taken after more than 120 days.

This study confirms the finding of Minke et al. [4] that there were significant differences in immunogenicity between the vaccines A and B in an experimental vaccination trial in laboratory dogs. Two vaccinations increased the numbers of dogs reaching approved test results in the present study (Table 1, Figure 1). However, the difference in performance between the two vaccines did not change with two vaccinations compared to one (there were no significant interaction between number of vaccinations and type of vaccine). The difference between the two vaccines can be caused by a true varying immunogenicity of the vaccines due to the virus strains, adjuvant used or the test system. The prescribed standardized FAVN test is using the CVS rabies virus strain as test virus and it was shown that use of homologous virus strains resulted in higher antibody titres in comparison to heterologous virus present in the vaccines [9].

Dogs vaccinated at an age less than 6 months or over 5 years of age had a higher failure rate than dogs between 6 months and 5 years. This is in concordance with findings of Mansfield et al. [2] and Kennedy et al. [3] that both showed a higher risk of lower antibody titres with increasing age as well as for dogs less than one year of age compared to adults. The higher risk of lower antibody titres in older dogs could be due to a reduced efficiency of the immune system with increasing age, however this reduced efficiency may not influence the antibody response [10,11]. The explanation for higher risk of lower antibody titres in younger dogs could be due to that the vaccine has been administered before the dog has reached immunocompetence [12].

In the present study significantly more dogs sampled at day 120-150 post vaccination reached approved test results than dogs sampled at day 151-180 (Tables 1, 2; Figure 1). An increasing proportion of dogs failing to reach the antibody response cut-off with increasing days from vaccination to sampling were also shown by Kennedy et al. [3]. Moreover, Jakel et al. [5] showed that dogs sampled up to 4 month after vaccination had a significantly higher chance of reach the antibody response cut-off than dogs sampled at later time-points independently if the dogs had been vaccinated once or twice. However, Kennedy et al. [3] arguments that this lower response may not relate to a lack of immune protection as the total immunoglobulin measure may be proportionately more accounted for by IgG as dog's iso-type shifts from an IgM response to an IgG as an immune response develops.

Medium-large and large-very large pure-bred dogs had less success reaching approved test results compared to very small-small pure-bred dogs vaccinated once. This difference was reduced when the dogs were vaccinated twice (Figure 1B). Kennedy et al. [3] showed that most failures were in larger breeds, but also some smaller breeds had important failure rates. Jakel et al. [5] could not find any differences in antibody response between breeds. We chose breed size as a factor and not specific pure breeds as such, and the high proportion of mixed breed dogs successfully reaching antibody response cut-off (Table 1) could be speculated to be either an effect of crossbreeding or that they might be of a small size, or a combination of both. It is well known that genetic variations across breeds are large, whereas within breed variation is much more limited. However, sampling cases from only one geographic location, i.e. Sweden, can cause false results for a particular breed due to a significant intra-breed genetic diversity between countries [13].

In concordance with Mansfield et al [2] and Jakel et al. [5] we could not find any differences in antibody response due to gender.

Based upon the results of the present study and the studies previously performed by others we would like to make the recommendations to vaccinate twice if the dog is of a larger breed.

## Conclusions

The probability of success of rabies vaccinations of dogs depends on type of vaccine used, number of rabies vaccinations, the breed size of the dog, age at vaccination, and number of days after vaccination when the antibody titres are tested. The need for a booster vaccination regimen is recommended for larger breeds of dog.

## Competing interests

The authors declare that they have no competing interests.

## Authors' contributions

LTB and BK initiated and designed the study. ER registered all data and AN performed all statistical calculations. LTB, BK and AN were all involved in the interpretation of results and drawing of conclusions, and have been equally active in writing this paper. All authors have read and approved the final manuscript.
